# Influence of Electronic Cigarettes on Selected Antibacterial Properties of Saliva

**DOI:** 10.3390/ijerph16224433

**Published:** 2019-11-12

**Authors:** Dominika Cichońska, Aida Kusiak, Barbara Kochańska, Jolanta Ochocińska, Dariusz Świetlik

**Affiliations:** 1 Department of Periodontology and Oral Mucosa Diseases, Medical University of Gdańsk, 80-204 Gdańsk, Poland; dominika.cichonska@gumed.edu.pl; 2 Department of Conservative Dentistry, Medical University of Gdańsk, 80-204 Gdańsk, Poland; bkochan@gumed.edu.pl (B.K.);; 3 Department of Biostatistics and Neural Networks, Medical University of Gdańsk, 80-211 Gdańsk, Poland; dariusz.swietlik@gumed.edu.pl

**Keywords:** e-smoking, electronic cigarettes, saliva

## Abstract

The aim of this study was to estimate changes in selected physicochemical properties of saliva collected from users of electronic cigarettes. *Methods:* The study population consisted of 120 patients (40 users of electronic cigarettes, 40 smokers of traditional cigarettes and 40 non-smokers). Laboratory tests included verification of saliva amount of lysozyme, lactoferrin and IgA. *Results:* Among e-cigarette users, statistically significant differences were observed in values of lysozyme and lactoferrin; however, no statistically significant differences for the IgA value were found. In the group of traditional cigarette smokers, statistically significant differences were observed among all tested parameters in relation to the control group. In relation to IgA, statistically significant differences were found between e-cigarette users and traditional cigarette smokers, to the disadvantage of the latter. *Conclusion:* Saliva of e-cigarette users showed changes of antibacterial properties in comparison to the control group and traditional cigarette smokers. Further longitudinal studies on larger study groups should be conducted in order to assess the effect of observed changes in the antibacterial properties of saliva on oral health.

## 1. Introduction

Saliva is a secretion constantly produced by large and small salivary glands, which primarily function is to provide homeostasis in the oral environment [1]. It consists mainly of water (about 99%), but also contains organic and inorganic substances that determine its physicochemical properties [2]. Individual elements occurring in saliva play a strictly defined role in the proper functioning of the whole organism, nourishing and protecting the surrounding tissues. Saliva glycoproteins moisturize the mucosa and provide protection to oral mucosa against mechanical damage. The presence of buffering bicarbonate and phosphate ions enables us to neutralize acids derived from food or that are a product of bacterial metabolism, which helps maintain an adequate value of saliva pH. There are also proteins present with enzymatic properties, which include salivary amylase. Saliva also contains antioxidants, including uric acid, glutathione, calalase, peroxidase, glutathione peroxidase and superoxide dismutase [3,4,5]. They are responsible for the neutralization of oxygen free radicals [6]. Moreover, many elements presenting antimicrobial activity are present in saliva. There are immunoglobulins A, lysozyme, lactoferrin, histamine and leukocytes. [2,7,8,9,10,11,12,13,14,15,16,17,18,19]. There are many factors that can influence saliva properties including genetic diseases and syndromes like Turner syndrome [20], or others such as general diseases (e.g., diabetes) or smoking [21]. Chemical compounds found in tobacco smoke can dissolve in saliva, leading to disorders in its biochemical composition [22].

Electronic cigarettes are gaining more and more popularity in the world, especially among young people, including those who have not smoked traditional cigarettes yet [23,24,25,26,27]. An electronic cigarette is a mechanical device that heats special inhalation solutions, giving the user an impression similar to smoking traditional cigarettes [28,29,30]. A few studies have proved that young e-cigarette users initially reported lower occurrence of negative side effects in comparison to smoking traditional cigarettes [31]. Initially, they were perceived as a less harmful alternative to tobacco smoking; however, they are beginning to arouse more and more controversy [28,29,32]. The aerosol generated during the usage of e-cigarettes leads to the appearance of adverse side effects in the oral cavity. Cytotoxic effects have been observed, leading to the death of oral epithelial keratinocytes [33,34] and periodontal fibroblasts [35]. The cytotoxic effect of electronic cigarettes was caused by the generation of oxidative stress and an increase in the release of proinflammatory cytokines [35,36]. 

The aim of our research was to assess whether electronic cigarettes have an influence on selected antibacterial properties of saliva, which can potentially affect the processes occurring in the oral environment.

## 2. Materials and Methods

### 2.1. Patient Population

In this study, 125 people participated (students of Medical University of Gdansk and young patients, who volunteered for a follow-up examination of periodontium and oral mucosa at the Department of Periodontology and Oral Mucosa Diseases), including 40 patients using e-cigarettes (e-cigarettes users), 40 patients smoking traditional cigarettes and 40 healthy patients that have not smoked cigarettes (non-smokers) as the control group. All groups included generally healthy people aged 20 to 30. Patients with diseases that might interfere with the condition of oral mucosa like diabetes, disorders of salivary secretion, oral mucosa diseases and people taking medications permanently and treated with antibiotics or steroid preparations in the last 6 months, as well as patients with periodontitis, were excluded from the research. E-cigarette users were using electronic cigarettes with small nicotine concentration for minimum 6 months vaping at least 50 times per day. Traditional cigarette smokers were smoking at least 10 cigarettes per day for a minimum of 6 months. There were no group of patients smoking both traditional and electronic cigarettes. The study was conducted in 2018–2019. The study protocol has been approved by the Ethics Committee of Medical University of Gdansk, Poland (NKBBN/161/2014). Ethical aspects of the research followed the World Medical Association Declaration of Helsinki. 

### 2.2. Saliva Collection

Mixed unstimulated whole saliva was collected from each patient studied. Saliva was collected into sterile silicone Corning-type test tubes in the morning hours, two hours after the last intake of food or drink. Unstimulated salivary samples were obtained by expectoration in absence of chewing movements.

The samples were clarified by centrifugation (2.000 × *g*; 10 min) and immediately stored at −20 °C for the later determination of IgA, lactoferrin and lysozyme.

### 2.3. Analysis of Saliva

The whole mixed unstimulated saliva was analyzed in the biochemical laboratory of Conservative Dentistry Department, Medical University of Gdańsk, Poland. The concentration of IgA, lactoferrin and lysozyme were analyzed by ELISA technology using commercially available kits (Sigma–Adrich, St. Louis, MO, USA). 

### 2.4. Statistical Analysis

The statistical analyses have been performed using the statistical suite StatSoft. Inc. (Tulsa, OK, USA) (2014), STATISTICA (data analysis software system) version 12.0. from www.statsoft.com and Excel. The significance of the difference between more than two groups was assessed with the F test analysis of variance (ANOVA) or Kruskal–Wallis. In the case of statistically significant differences between two groups, post hoc tests were utilized (Tukey test for F or Dunn for Kruskal-Wallis). In all the calculations, the statistical significance level of *p* < 0.05 has been used.

## 3. Results

Table 1 presents the value of IgA, lysozyme and lactoferrin levels on unstimulated saliva among e-cigarette users, cigarette smokers and the control group. 

The value of IgA concentration in e-cigarette users was 201 ± 118 µg/mL (range 12.0–560.0, Me = 169.0), the result in the group of traditional cigarette smokers was 164.7 ± 95 µg/mL (range 16.0–332.0, M = 157.5) and in control group it was 515.8 ± 430 µg/mL (range 36–2182, Me = 399.0). Statistical analysis presented significant differences between e-cigarette users and traditional cigarette smokers to the disadvantage of cigarette smokers. Statistically significant differences were also observed between traditional cigarette smokers and the control group (*p* < 0.05). 

The value of lysozyme concentration in e-cigarette users was 1.7 ± 0.9 µg/mL (range 0.2–3.8, Me = 1.6), the result in the group of traditional cigarette smokers was 1.8 ± 1.4 µg/mL (range 0.3–6.1, Me = 0.3–6.1, Me = 1.4) and in the control group it was 6.5 ± 4.8 µg/mL (range 1.3–22.1, Me = 4.8). Statistical analysis presented statistically significant differences between e-cigarette users and the control group, as well as between traditional cigarette smokers and the control group (*p* < 0.05).

**Figure 1:**
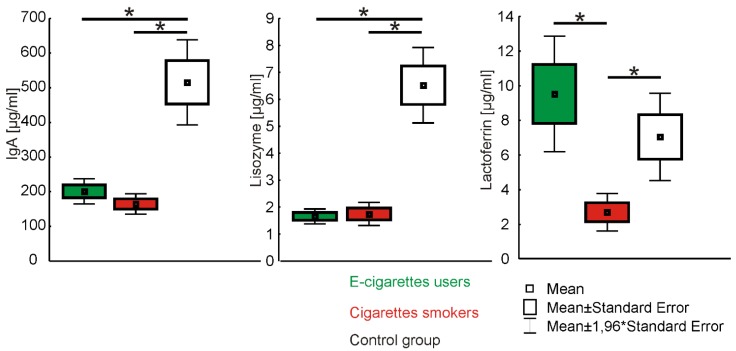
Mean values on unstimulated saliva IgA, lisozyme and lactoferrin concentration in e-cigarette users, cigarette smokers and the control group. *statistically significant values.

The value of lactoferrin concentration in e-cigarette users was 9.5 ± 10.6 µg/mL (range 0.1–40.4, Me = 7.1), the result in the group of traditional cigarette smokers was 2.7 ± 3.5 µg/mL (range 0.3–15.7, Me = 1.4) and in the control group it was 7.0 ± 8.8 (range 1.1–61.7, Me = 5.6). Statistical analysis proved statistical significance in both e-cigarette users and cigarette smokers in comparison to the control group (*p* < 0.05) (Figure 1).

## 4. Discussion

Saliva is the first biological fluid that has contact with both tobacco smoke and aerosol e-cigarette and, therefore, is an important line of defense against occurring harmful factors. Tobacco smoking leads to numerous adverse side effects in the oral cavity, including changes in the antibacterial properties of saliva [3,37]. 

Immunoglobulin A is the only antibody that is actively secreted into saliva, and its concentration in saliva among people with no pathological changes in the periodontium is relatively low. However, it increases in the presence of periodontitis, constituting a defense mechanism against antigenic stimuli derived from dental bacterial biofilm of dental plaque. IgA may also occur as a specific immunoglobulin against *A. actinomycetemcomitans*—it has been found in patients with diagnosed refractory periodontitis [2]. Lowering the level of IgA in saliva may lead to a weakening of the specific immune response and cause earlier development of more severe periodontitis [8].

In previously conducted studies among traditional cigarette smokers, a decrease in the level of immunoglobulin A in saliva in comparison to non-smokers was observed [38]. This was also confirmed by research carried out by Barton et al. and Bennet, which presents a reduction in IgA levels in groups of tobacco smokers [8,39]. However, studies conducted by Kibayashi et al. did not prove statistically significant differences in the level of IgA in saliva between tobacco smokers and non-smokers [40]. In our research, it was observed that the tobacco smokers’ level of salivary IgA was statistically significantly lower in comparison to the values in both the control group and the group of e-cigarette users. IgA content in the saliva of e-cigarette users was not statistically significant lower compared to the control group. This indicates that electronic cigarettes compared to traditional cigarettes have less effect on IgA concentration in saliva. 

Lysozyme present in saliva mainly originates from both large and small salivary glands; however, a little amount comes from leukocytes and fluid from the gingival pockets. This is an enzyme that causes lysis of bacterial cell walls. Despite the presence of antibacterial activity, it also presents antiviral and antifungal activity [9,10,11,12,13].

In our research, the level of lysozyme in the group of traditional cigarette smokers compared to the control group was lower, as confirmed by studies conducted by Rudney [39]. Among e-cigarette users, in comparison to the control group, there was also a decrease in lysozyme levels in saliva, which may indicate a similar effect of both traditional and electronic cigarettes on the content of lysozyme in saliva. 

Lactoferrin is an iron-binding glycoprotein secreted by the serous cells of the small and large salivary glands. Its elevated level indicates the currently occurring inflammatory processes. Similarly to lysozyme, the antibacterial, antiviral and antifungal activities of lactoferrin have been proven. Lactoferrin has also immunomodulatory and anti-inflammatory effects on the oral environment [14,15,16,17,18,19].

Our research has proven that the level of lactoferrin in the saliva of traditional cigarette smokers was lower than in the control group. This corresponds to the results obtained by Nishida et al., Kibayashi et al. and Rudney, where lactoferrin levels also tended to decrease among tobacco smokers [39,40,41]. However, in the group of e-cigarette users, elevated levels of lactoferrin in saliva were found. This provides different affects of electronic cigarettes and smoking on the content of lactoferrin in saliva.

There is a large number of already conducted studies on the effect of traditional cigarettes on the antibacterial properties of saliva [2,38,39,40,41,42]; however, no such studies have been carried out for e-cigarette users so far. Therefore, our obtained results that prove changes in the oral ecosystem, particularly elements related to the bacterial defense system, are the first to regard this issue and they indicate the need for further long-term research.

## 5. Conclusions

Saliva of e-cigarette users showed changes of antibacterial properties in comparison to the control group and traditional cigarette smokers.

Further longitudinal studies on a larger study group should be conducted in order to assess the effect of observed changes in the antibacterial properties of saliva on oral health.

## Figures and Tables

**Table 1:** Mean values on unstimulated saliva IgA, lisozyme and lactoferrin concentration in e-cigarette users, cigarette smokers and the control group.

Groups		IgA [µg/mL]			Lisozyme [µg/mL]			Lactoferrin [µg/mL]	
	Mean ± SD	Range	Me	Mean ± SD	Range	Me	Mean ± SD	Range	Me
E-cigarette users (n = 40)	201.1 ± 118	12.0–560.0	169.0 ^a^	1.7 ± 0.9	0.2–3.8	1.6 ^c^	9.5 ± 10.6	0.1–40.4	7.1 ^e^
Cigarette smokers (n = 40)	164.7 ± 95	16.0–332.0	157.5 ^a,b^	1.8 ± 1.4	0.3–6.1	1.4 ^d^	2.7 ± 3.5	0.3–15.7	1.4 ^f^
Control group (n = 40)	515.8 ± 430	36.0–2182.0	399.0 ^b^	6.5 ± 4.8	1.3–22.1	4.8 ^c,d^	7.0 ± 8.8	1.1–61.7	5.6 ^e,f^

Legend: ^a, b, c, d, e, f^—testify to statistically significant values; ^a-a, b-b, c-c, d-d, e-e, f-f^—groups with statistical significance; *p* < 0.05 for ^a-a, b-b, c-c, d-d, e-e, f-f^, Mean—mean values, SD—standard deviation, Me—median.
